# Spatial analysis of risk factors related to colorectal cancer in Iran: An ecological study

**DOI:** 10.1002/hsr2.70120

**Published:** 2024-10-06

**Authors:** Zahra Montaseri, Hossein Kargar, Mehdi Sharafi, Sima Afrashteh

**Affiliations:** ^1^ Department of Infectious Diseases, School of Medicine Fasa University of Medical Sciences Fasa Iran; ^2^ School of Medicine Fasa University of Medical Sciences Fasa Iran; ^3^ Tobacco and Health Research Center Hormozgan University of Medical Sciences Bandar Abbas Iran; ^4^ Department of Biostatistics and Epidemiology, Faculty of Health and Nutrition Bushehr University of Medical Sciences Bushehr Iran

**Keywords:** colorectal cancer, GIS, Iran, spatial analysis

## Abstract

**Background and aims:**

Colorectal cancer is the third most common cancer worldwide, accounting for 10% of cancer deaths. Therefore, this study was performed with the aim of spatial analysis of risk factors for colorectal cancer in Iran.

**Method:**

This study was conducted ecologically using STEPS information (The WHO Stepwise Approach to NCD Risk Factor Surveillance) in Iran. To analyze the data, the researcher used cluster analysis and Geographically Weighted Regression methods with the help of ArcGIS version 10.

**Results:**

The results of OLS analysis showed that there was a significant relationship between tobacco consumption (*B* = 0.571, *p*‐value = 0.044) and smoking (*B* = 0.772, *p*‐value = 0.010) and the incidence of colon cancer (CC). There was also a significant relationship between abdominal obesity and the incidence of rectal cancer (RC) (*B* = 0.061, *p*‐value = 0.027).

**Conclusion:**

This study showed that (CC) high‐risk areas are located in central and northern parts of Iran, and the significant risk factors related to CC and RC were found to be tobacco use, cigarette smoking, and abdominal obesity. These findings are helpful to inform policymakers to plan screening services to reduce CC and RC, especially in high‐risk populations.

## 1 INTRODUCTION

Cancer is one of the main health problems and the third leading cause of death in the world. Colorectal cancer, the third main cancer worldwide, accounts for 10% of cancer deaths^1^ Colorectal cancer, occurring in the colon or rectum, is classified into one group because of its common characteristics. The wall of the colon and rectum is made up of several layers. Colorectal cancer begins in the inner layer (mucosa) and can spread to another layer or layers. Therefore, the cancer cells located in the wall can grow in the blood vessels or lymphatic vessels and get transmitted to the lymph nodes near or farther in the body. It usually takes 10–15 years from the time abnormal cells begin to grow to the development of colorectal cancer. As a result, regular screening can prevent the incidence and mortality of this cancer.^2^ According to the GLOBOCAN (Global Cancer Incidence) reported in 2012, the number of new cases and deaths due to this cancer were estimated at 1,36,1000 and 694,000, respectively.^2^, ^3^ The World Health Organization predicts a 77% increase in new cases and an 80 percent increase in colorectal cancer deaths by 2030.^4^, ^5^

Most colorectal cancers occur worldwide in industrialized countries; however, in less developed countries, the incidence is rapidly increasing due to the adoption of the Western lifestyle.^6^ The highest incidence is in Australia and the lowest is in West Africa.^3^ Before 1900, colorectal cancer was relatively uncommon in the United States, but along with economic development, its incidence has increased dramatically over the past century.^6^ Research shows that colorectal cancer mortality has increased in Asia over the past 10 years.^7^ Japan has the highest incidence of colorectal cancer, especially in men (41.7 cases per 100,000 people), however, as it has been one of the first countries to implement a screening program since 1992, the mortality rate in this country is lower than in Europe. Second to Japan is Europe, with the highest incidence and mortality rates as one of the affected regions.^8^, ^9^

The standard incidence of this cancer in Iran has been reported as 7 cases per 100,000. It is the fourth most common cancer in Iran. With a mortality rate of 1.98 per 100,000, this type of cancer comprises about 13% of the deaths of gastrointestinal cancers and 5.3% of deaths from other causative factors.^10^ The mortality rate of colorectal cancer is steadily rising in Iran. As the population ages, the death rate increases as well.^11^ Risk factors responsible for colorectal cancer include a diet without fruits and vegetables, excessive consumption of red meat and saturated fats, drinking alcohol, lack of physical activity, smoking tobacco, overweight, family history, and age over 50 years.^8^, ^12^ So, the number of cases that can be prevented by adopting a healthy lifestyle is estimated from 66% to 75%.^13^

The incidence of cancer has increased in both sexes in Iran; however, men are about two times more likely susceptible than women.^14^ In addition, women in the lower age group face an increased risk of developing this type of cancer.^15^ In Iran, the northern and northwestern provinces of Iran such as Ardebil have the highest and the southwestern provinces such as Kokiluyeh Booyer Ahmad have the lowest incidence rate of cancer in men. The highest cancer incidence rate among women belongs to Semnan and the lowest to Hormozgan and Kohkiluyeh Booyer Ahmad Provinces.^14^ The distribution of colorectal cancer and its causative factors in various geographical areas is well documented. This topic provides the development of a scientific field called health geography, which discusses the effects of various factors on human health.^16^

Programs to control cancers including prevention, screening, timely treatment, and relief services are the system of collection, recording, and analysis of new cases of cancer. The analysis of these data besides reports of authorities and scientific research will enlighten the way of the disease outbreak, the disease progression, and different types of cancers in various areas. The cancer registration system is the main tool for managing and controlling cancers. This important source of information is essential not only for epidemiological studies of the disease but also for planning and predicting events, assessing the accuracy and consistency of the research, and the impact of the required interventions. In other words, programs for screening and evaluating the efficacy of research, and cancer prevention can be provided following data collection at the cancer database. As a result, without a cancer registration system, it is clear that a control service is like a shot in the dark.

It should be noted that the cancer registration system is an essential coherent system that can only be effective when cancer reports are continuously recorded in the country. Moreover, the continuous system of cancer registration would facilitate the evaluation, and examination of the effects of natural and planned interventions. Therefore, this study was conducted to determine the factors related to the distribution of colorectal cancer in the provinces of Iran.

## 2 MATERIALS AND METHODS

### 2.1 Study population and data gathering

This ecological study was performed using STEPS information in Iran. The step‐by‐step approach of World Health Organization has three levels of data collection to surveil the risk factors for non‐communicable diseases. Its three executive steps comprise questioning through a questionnaire, completion of information with physical measurements, and laboratory tests: Step one: questioning through a questionnaire: Questions on tobacco consumption, diet, physical activity, history of high blood pressure and diabetes, and such questions as wearing safety belt of the front seat and health insurance coverage; step two: Physical measurements: measurements of blood pressure, body mass index, and waist circumference; step three: Laboratory tests: fasting blood glucose and blood lipids tests.

Colon cancer (CC) and rectal cancer risk factors were studied from the first two basic steps (ecological data and behavioral, and physical measurements) during seven periods. The data were recorded in the VIZIT system. Laboratory measurements (step three) were also included in the study at 3‐year intervals (from 2004 to 2007). CC and RC information was also extracted based on the national cancer registration system from each province. The first period of these studies was performed in 2004 with a sample size of 89404 people and was implemented by multistage cluster sampling at the provincial and national levels. The second to fifth periods of these studies were carried out with the same sample size and method, related to health requirements of the country. In 2011, the sixth period of the “National Investigation of the Risk Factors of Noncommunicable Diseases” was performed using the Multistage Cluster Random Sampling method with a sample size of 12,000 people. The risk factors related to non‐communicable diseases were used in this study, including smoking, physical inactivity, dairy consumption, fish, salt, fast food, fruits and vegetables, etc. The latest information recorded in this study was related to 2016.

Fifty clusters containing five age groups (15–24, 25–34, 35–44, 45–55, 56–64) were selected from each province. Each cluster consisted of 10 people of both sexes (2 people from each group). Overall, 1000 urban and rural populations from each province and 30,000 people from the whole country entered in these studies. Therefore, this survey covers 60% of the population 64–15 years old. The quality control of all measurements is carried out through periodic control by provincial observers. we used ecological data from all provinces in Iran. This type of data focuses on whether a species is present or absent in a particular area. It is often used in monitoring programs to track changes in species distribution patterns.

### 2.2 Statistical analyses

Hot spot analysis was conducted to identify high‐risk locations for colon and rectal cancers. To assess the relationship between covariates and the incidence of these cancers, we utilized the Geographically Weighted Regression (GWR) model. Before the GWR model was implemented, an Ordinary Least Squares (OLS) model was employed to select potential predictor variables. Only those variables with a significance level of less than 0.25 were included in the GWR model, meaning variables with a p‐value greater than 0.25 were deemed not significant enough for inclusion.

The primary goals of using the OLS model were twofold: first, to identify the key variables that explain the variation in the incidence of colon and rectal cancers, and second, to address potential confounders. Subsequently, the GWR model was applied to examine how these significant variables varied geographically, helping to identify spatial patterns in the relationship between these covariates and cancer incidence. Based on this analysis, we were able to predict the incidence of colon and rectal cancers across the provinces. In all analyses, a significance level of less than 0.05 was considered.

## 3 RESULTS

**Figure 1:**
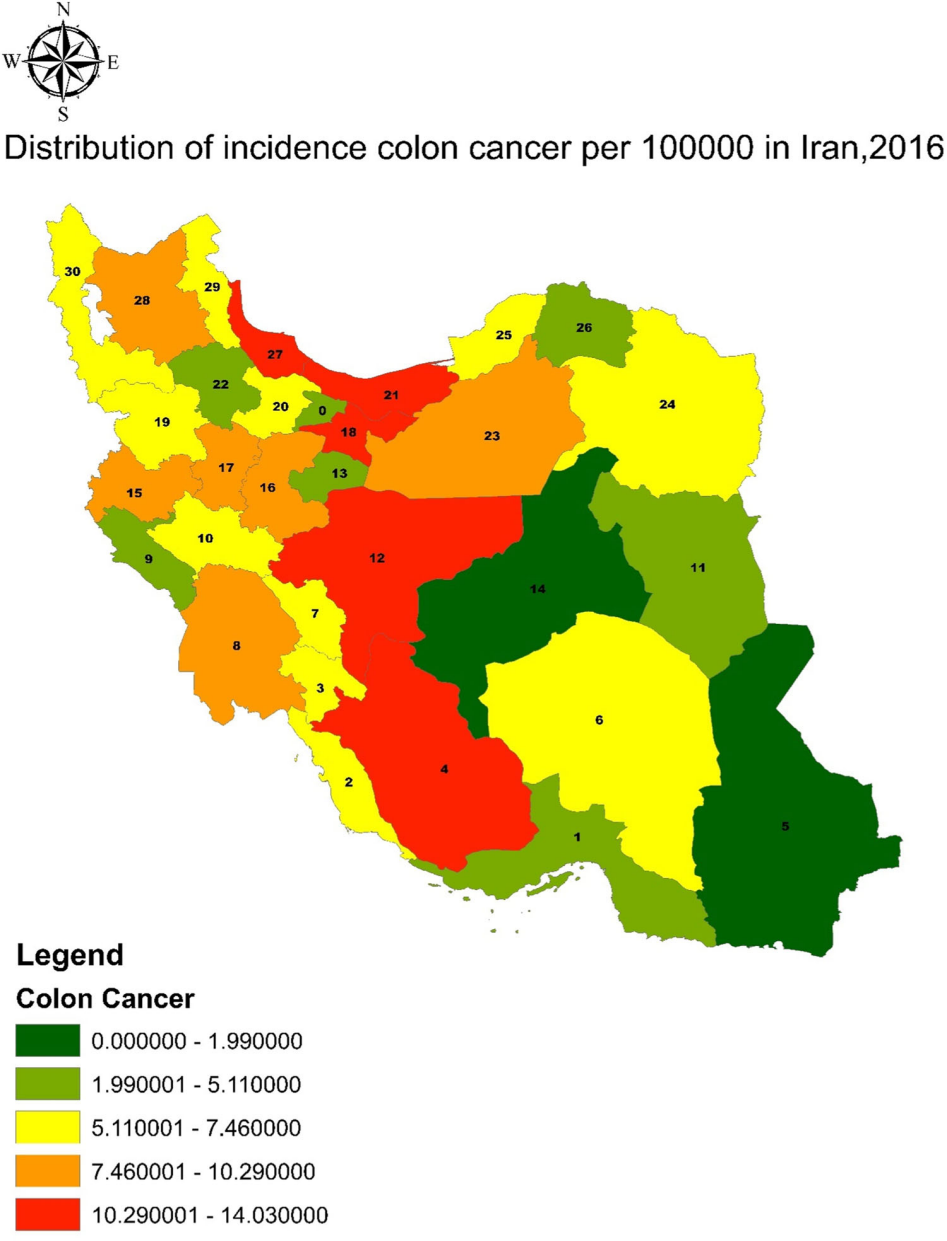
Distribution of incidence Colon cancer/100,000 2016.

**Figure 2:**
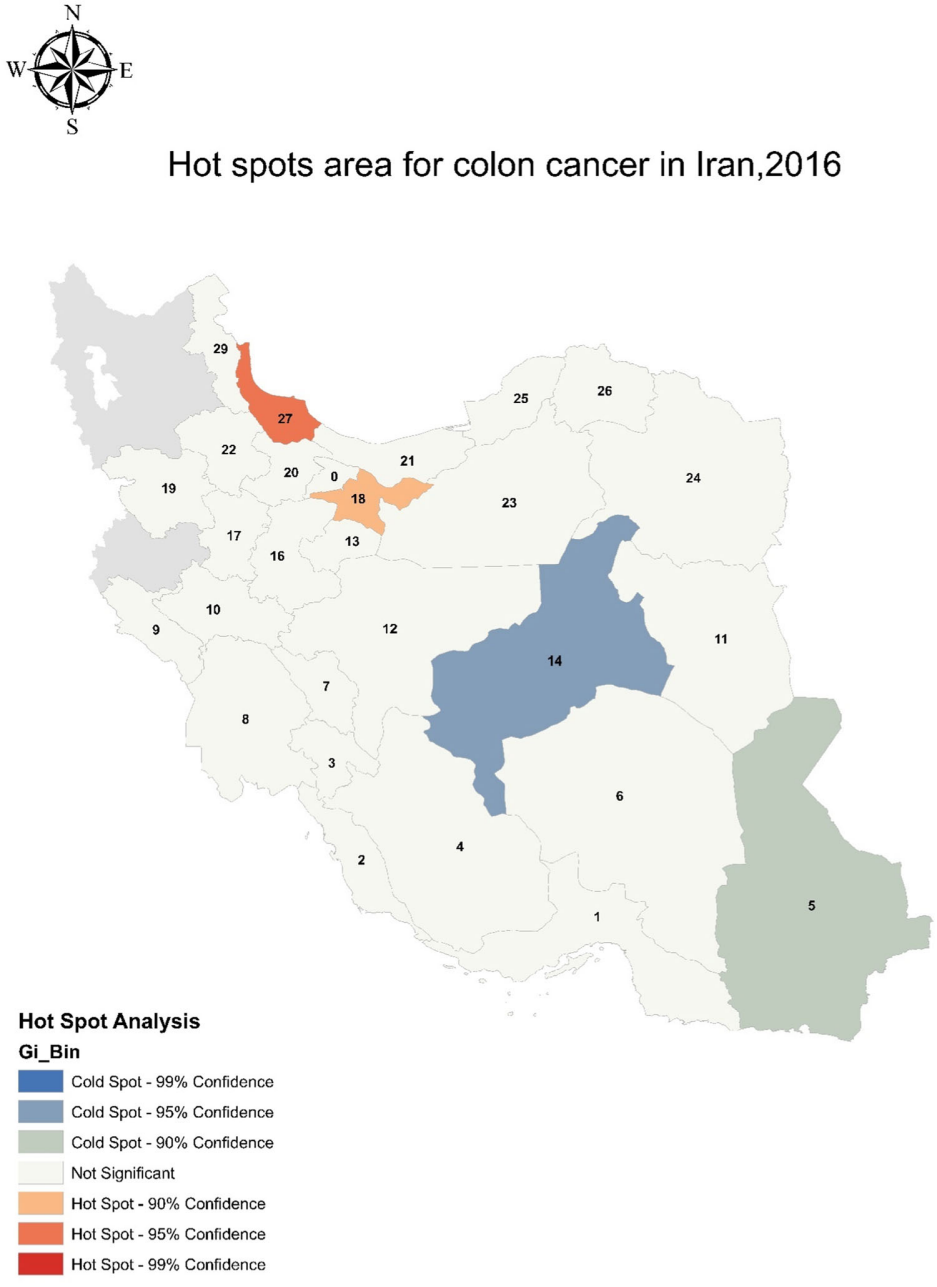
Hot spots area for colon cancer in Iran, 2016.

The purpose of this study was to analyze the risk factors for colorectal cancer in Iran using GIS. In this regard, the information about the prevalence of each factor was first calculated by provincial separation and then presented in the descriptive and inferential findings. Table 1 presents descriptive information on the amount or the percentage of each variable in the provinces of Iran. Figure 1 shows the dispersion map of CC per 100,000 people in the provinces of Iran, and Figure 2 denotes the analysis of hot spots. These figures show that the central and northern provinces are at the highest risk and incidence of CC. Mazandaran, Gilan, Tehran, Isfahan, and Fars provinces have the highest incidence, while the lowest incidence was observed in Yazd, Sistan, and Baluchistan provinces.

**Table 1:** Descriptive information of the amount or the percentage of each variable in the provinces of Iran.

Province	Code	Cigarette	Alcohol	Hemoglobin	Fish	Salt	Cholesterol	Physical Activity	Fast Foods	Vegetables	Fasting Blood Sugar	Obesity	Fat Around the waist	Tobacco	Fruits	Incidence of Rectum Cancer per100,000	Incidence of Colon Cancer Per100,000
		Cigarette smoking in terms of percentage	Drinking alcohol in terms of percentage	Mean in terms of percentage	Consumption of good fish in terms of percentage	Excessive consumption of salt in terms of percentage	Mean in milligram in Deciliter	Prevalence in terms of percentage	Consumption of Fast foods in terms of percentage	Consumption of vegetables in terms of percentage	Mean in milligram in Deciliter	Obesity in terms of percentage	Prevalence of fat around the waist in terms of percentage	Consumption of tobacco in terms of percentage	Consumption of fruits in terms of percentage	New cases per 100,000	Tab
Qazvin	20	12.61	24.13	5.55	2.72	12.76	161.48	46.61	21.36	38.38	89.3	23.61	62.66	20.84	45.93	2.16	7.20
Bushehr	2	7.99	9.21	5.62	56.63	5.20	166.05	34.80	12.34	66.05	98.73	18.5	69.33	20.71	23.62	2.62	6.02
Hormozgan	1	6.84	6.07	5.61	55.81	5.45	164.22	30.15	18.34	27.88	96.68	11.71	53.18	17.47	8.96	1.67	4.4
Fars	4	9.02	10.81	5.59	11.42	6.39	157.84	47.78	19.93	50.31	96.21	19.83	56.56	17.07	17.67	4.26	11.75
Kokiluyeh va Bouyer Ahmad	3	7.91	8.66	5.41	13.69	3.88	151.48	63.31	16.11	60.66	91.04	20.97	53.52	16.71	30.47	2.34	5.88
Markazi	16	13.5	10.53	5.59	1.91	11.71	157.6	39.52	15.22	47.88	95.24	18.21	58.42	16.17	22.61	2.86	9.68
Hamadan	17	10.19	9.60	5.56	4.31	11.2	161.04	39.77	12.2	39.52	94.83	18.85	57.04	15.51	22.71	2.22	10.29
Azarbayejan Qarbi	30	13.48	12.88	5.62	2.15	20.79	167.26	55.87	9.58	48.21	96.71	25.83	51.34	15.37	16.11	1.96	6.23
Mazandaran	21	9.61	12.61	5.57	7.99	4.06	170.62	42.29	13.55	50.99	100.58	29.63	59.84	15.31	29.19	3.44	11.47
Lorestan	10	9.25	4.55	5.54	6.43	25.77	165.38	35.35	11.34	39.45	93.42	20.83	53.4	15.18	17.60	1.93	7.15
Systan va Baluchestan	5	6.56	2.14	5.67	22.06	2.06	155.8	31.06	5.32	20.80	94.44	11.81	56.11	14.91	2.81	0.75	1.99
Esfahan	12	10.01	11.13	5.58	5.9	6.79	161.42	44.43	15.98	38.46	96.8	19.89	61.07	14.87	21.96	3.84	11.22
Alborz	0	12.27	7.73	5.60	3.04	12.18	160.71	44.66	14.27	28.38	98.95	22.97	58.03	14.81	6.70	1.8	5.11
Chahar Mahal va Bakhtyari	7	11.36	6.19	5.54	6.93	3.15	161.00	41.70	9.58	47.31	91.43	18.03	48.94	14.77	26.10	2.61	7.46
Yazd	14	8.09	7.26	5.74	4.28	4.93	162.98	44.57	23.68	28.48	97.78	22.30	68.65	14.68	12.06	5.03	14.12
Kordestan	19	12.18	14.68	5.52	1.66	20.02	157.95	39.19	7.40	26.55	91.35	22.12	54.66	13.51	12.73	1.86	6.25
Khorasan Razavi	24	6.18	7.86	5.63	3.48	12.76	164.19	45.56	13.61	38.85	95.74	18.81	53.24	13.19	13.75	2.56	7.39
Zanjan	22	9.94	9.97	5.54	4.13	15.65	157.63	42.53	12.56	54.17	93.57	19.87	57.35	13.17	19.37	1.44	4.67
Tehran	18	9.98	7.71	5.53	5.70	9.90	165.70	41.21	15.83	44.18	100.31	19.70	63.56	12.49	20.01	3.83	12.48
Ardabil	29	11.20	5.01	5.54	11.47	21.29	158.42	51.93	18.33	29.92	90.41	26.82	55.66	12.20	8.33	2.09	6.09
Guilan	27	10.57	8.65	5.55	24.81	6.95	162.83	53.01	8.08	48.78	101.31	24.78	60.55	11.93	15.37	4.78	14.03
Khouzestan	8	8.94	4.54	5.70	29.41	8.72	166.42	39.30	19.36	50.25	99.92	23.93	59.61	11.91	11.91	3.27	9.70
Azarbayejan Sharqi	28	10.90	4.63	5.55	2.09	15.97	163.45	59.30	12.49	47.66	96.55	21.70	64.69	11.89	16.28	2.42	9.90
Khorasan Shomali	26	4.63	6.40	5.50	2.55	6.7	165.30	43.50	11.68	48.44	95.72	17.49	55.52	11.25	22.31	1.16	3.80
Keman	6	6.93	11.21	5.66	5.90	7.31	159.42	31.50	16.23	24.50	96.52	15.64	57.09	10.96	15.37	2.85	6.79
Kermanshah	15	9.06	4.37	5.63	4.19	20.39	153.13	67.04	6.81	32.75	91.66	19.99	54.51	10.65	14.03	2.37	9.07
Golestan	25	4.99	14.40	5.77	6.75	11.98	163.17	32.80	15.75	45.49	104.28	25.66	53.98	10.27	22.10	1.93	6.16
Semnan	23	8.68	4.35	5.79	4.00	12.95	162.57	49.56	17.60	54.98	101.44	25.73	67.68	9.80	24.56	2.91	10.12
Khorasan Jonoubi	11	4.85	1.73	5.57	4.18	8.64	162.44	40.33	14.77	43.43	89.50	16.87	51.16	7.22	17.63	1.88	4.86
Eilam	9	4.97	3.37	5.50	9.09	26.80	164.20	42.29	6.52	37.45	94.79	12.84	58.75	6.70	8.42	1.50	4.72
Qom	13															1.80	4.24

**Figure 3:**
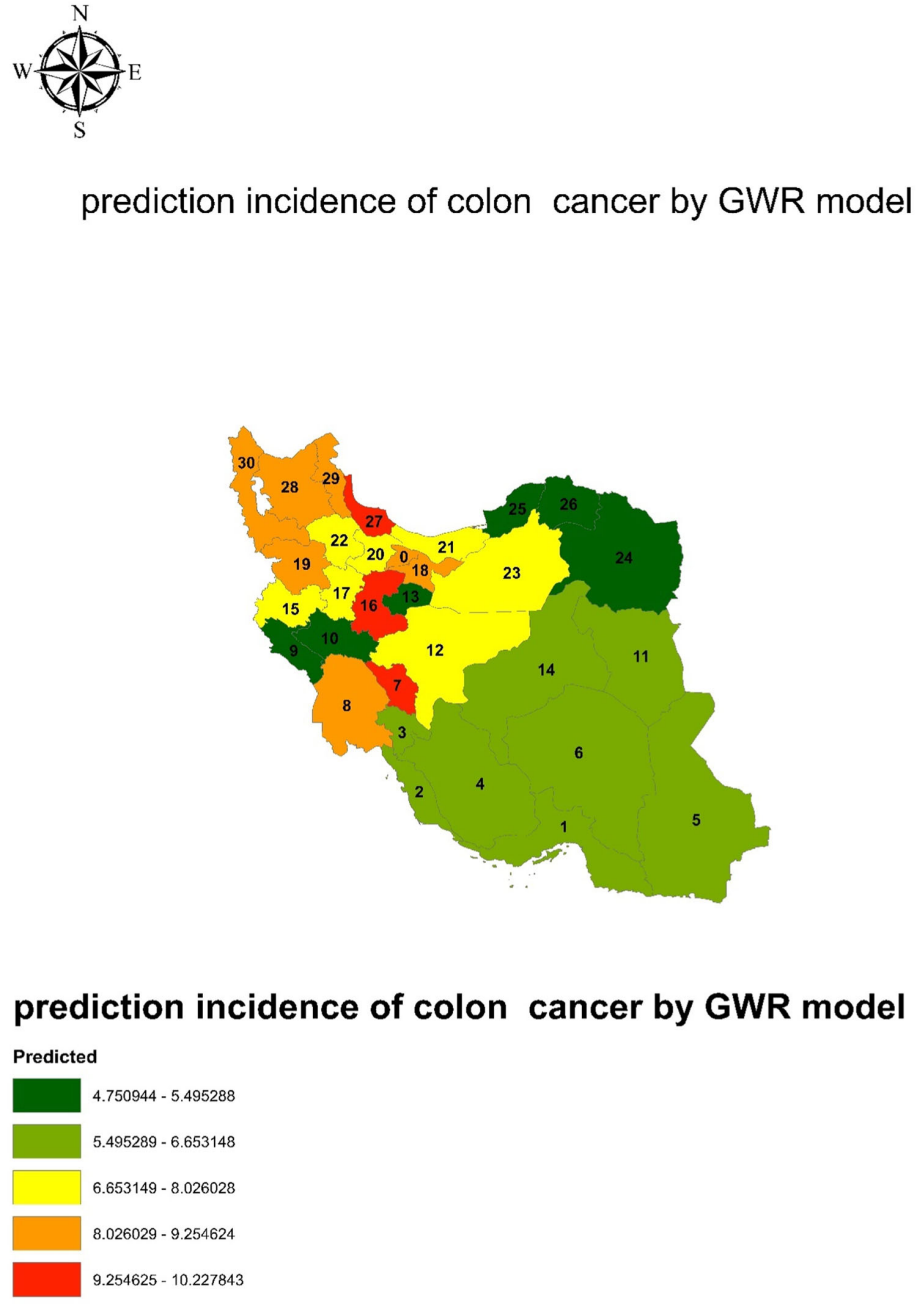
Predicted incidence of colon cancer by GWR model and fitted indexes.

Table 2 shows the results of the OLS analysis. Accordingly, tobacco use (*B* = 0.571, *p*‐value = 0.044 and cigarette smoking (*B* = 0.772, *p*‐value = 0.010) had a significant correlation with the incidence of CC (*p* < 0.05). However, for modeling and prediction of CC and RC in provinces, the variables tobacco use, salt, and fish consumption were included in the GWR model that possessed a *p*‐value ≤ 0.25 in the OLS model. Figure 3 shows the prediction of the GWR model and the indices of model fitness. GWR model resulted that the northwest provinces, including Eastern and Western Azarbaijan, Gilan, Tehran, Qazvin, and Khuzestan have the highest incidence rates of CC, marked in red. However, Khorasan province has the least incidence rate of CC, marked in blue.

**Table 2:** Ordinary least squares regression results (OLS) for accessing relationships between predictor's variables and colon cancer.

		Regression coefficient	Standard error	*p*‐value
Consumption of foods	Consumption of fruits	0.09	0.139	0.515
	Fast food consumption	−0.02	0.162	0.869
	Salt	−0.077	0.106	0.301
	Fish	0.061	0.066	0.263
	Consumption of vegetables	0.049	0.085	0.547
Body condition	The abdominal obesity	0.027	0.134	0.790
	Obesity	0.007	0.213	0.951
	physical activity	0.025	0.086	0.639
consumption of medicine	Alcohol	0.064	0.23	0.706
	Tobacco	**0.571**	0.343	**0.044**
	Cigarette	**0.772**	0.381	**0.010**
Blood Factors	Cholesterol Fat	0.063	0.153	0.525
	hemoglobin	−2.305	4.522	0.413
	Fasting blood glucose	0.0032	0.013	0.617

*Note*: Dependent variable: Incidence of colon cancer. The bold values represent variables with a significance level of less than 0.05.

**Figure 4:**
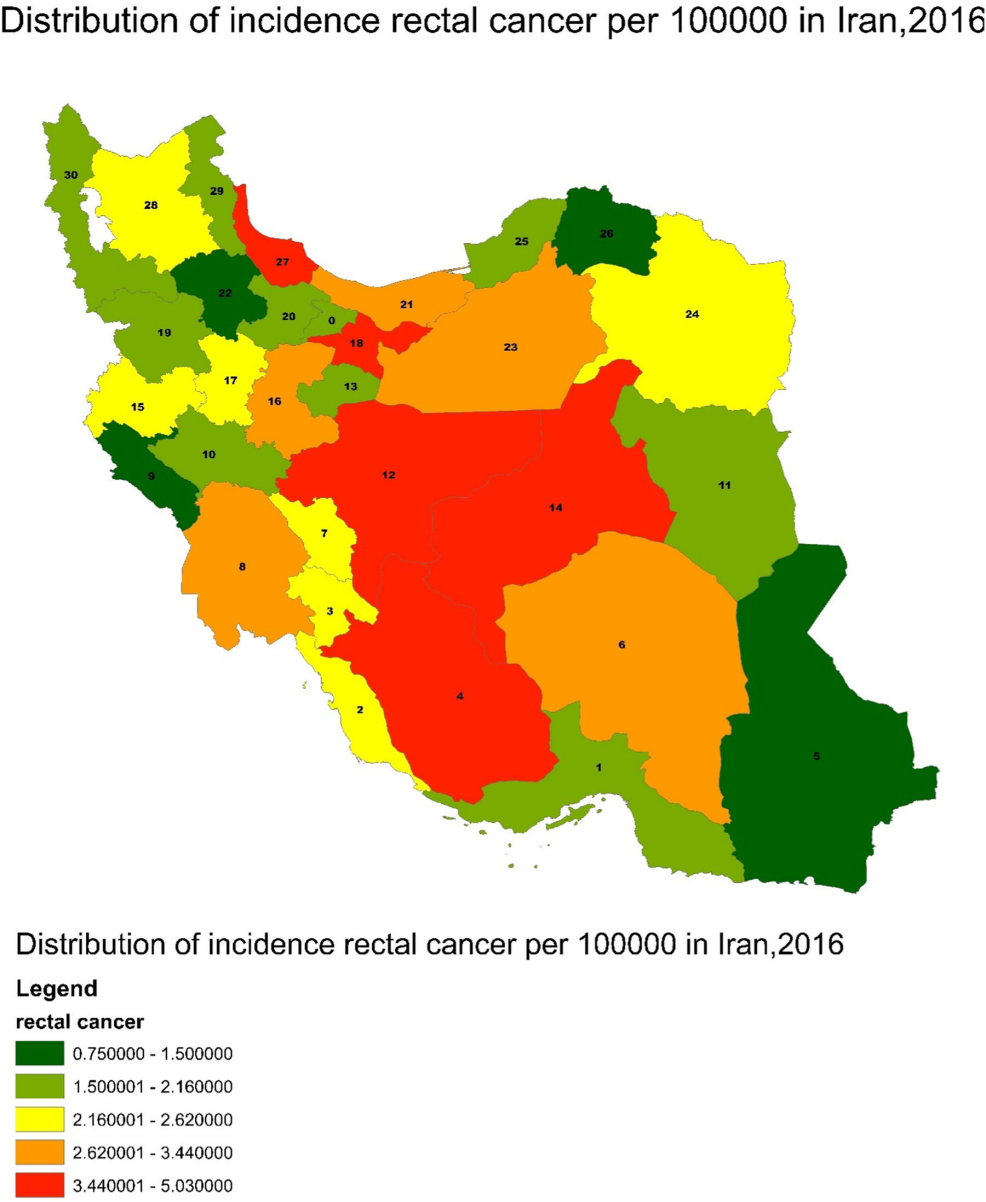
Distribution of incidence rectal cancer/100,000 in Iran, 2016.

**Figure 5:**
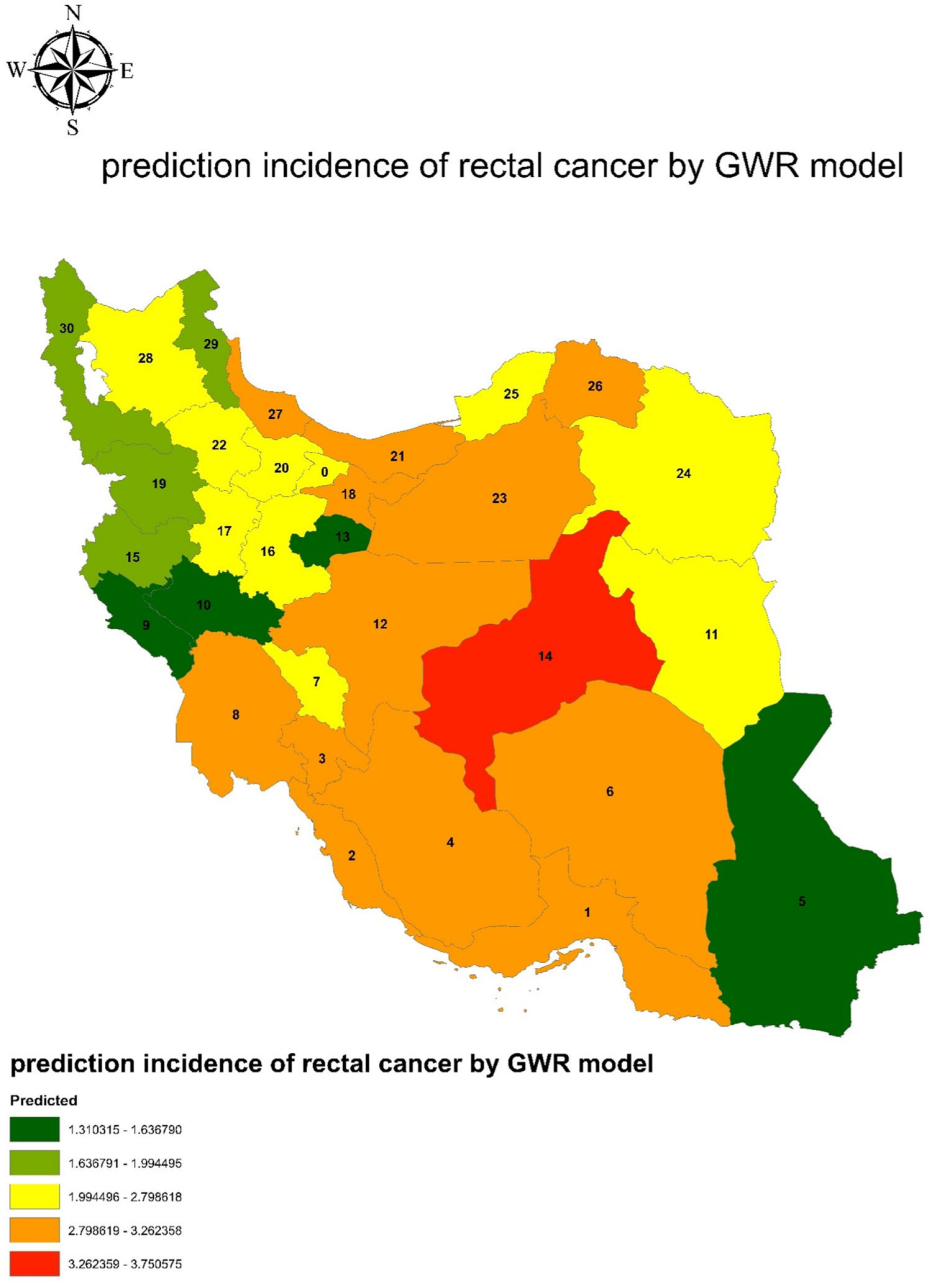
predicted incidence of rectal cancer by GWR model and fitted indexes.

Figure 4 show the distribution of incidence rectal cancer per 100,000 person in Iran, 2016. To examine the relationship between the underlying variables and rectal cancer, the researcher used the OLS model as well. Based on the results of this model (Table 3), abdominal obesity has a significant relationship with rectal cancer (*B* = 0.061, *p*‐value = 0.027). However, for the GWR model, other variables such as eating fast foods and salt, which had a significant level less than 0.25, were also included. Figure 5 shows the result of the GWR prediction and model indices based on independent variables. The color spectrum of dark green to red is indicative of the rectal cancer prediction in the order of low to high risk.

**Table 3:** Ordinary least squares regression results (OLS) for accessing relationships between predictor's variables and rectal cancer.

Variable		Regression coefficient	Standard error	*p*‐value
Consumption of foods	Consumption of fruits	−0.034	0.04	0.326
	Fast food consumption	0.074	0.051	0.069
	Salt consumption	−0.060	0.032	0.062
	Fish consumption	0.002	0.02	0.891
	Vegetables consumption	0.008	0.025	0.664
Body condition	Abdominal obesity	**0.061**	0.041	**0.027**
	Obesity	0.024	0.064	0.530
	Physical activity	0.019	0.026	0.323
Drug using	Alcohol consumption	0.037	0.07	0.500
	Tobacco use	0.082	0.106	0.341
	Cigarette smoking	0.113	0.118	0.211
Blood Factors	Cholesterol Fat	0.015	0.046	0.633
	Hemoglobin A_1_c	−1.292	1.363	0.176
	Fasting blood glucose	0.003	0.011	0.512

*Note*: Dependent variable: Incidence of rectal cancer. The bold values represent variables with a significance level of less than 0.05.

## 4 DISCUSSION

CC is considered significant cancer in the world that inflicts about 10% of the world's population.^17^ The prevalence of cancer has a different geographical distribution. This cancer is the third most common cancer with an incidence of 7% in Iran.^18^ This study was a spatial analysis of the development of CC to determine some risk factors attributed to this disease using the geographical weight regression model in Iran.

This research used a hot spot method and showed the highest prevalence of disease in the northern and central regions of Iran which complies with previous reports using hot spot models and joint components.^19^, ^20^, ^21^ In addition, the highest incidence of cancer in Mazandaran, Guilan, Tehran, Isfahan, and Fars provinces are similar to the results of Mahaki et al.'s study using the model of spatial‐time components.^22^ Risk factors affecting CC identified in this research consisted of age above 50 years, gender (male more than female), socioeconomic status, education level, diet, and lifestyle. These factors are also outlined in former studies.^17^, ^18^, ^23^, ^24^ Pourhoseingholi et al. suggested that socioeconomic factors such as unemployment and household income are significant risk factors in the northern and central regions of Iran.^18^ In addition, lifestyle and dietary habits are known as the main risk factors responsible for CC.^25^ Social and economic factors can significantly affect dietary habits.^3^, ^12^ For example, in a study conducted in the neighborhoods of Tehran, Iran, low‐income and low socioeconomic status were recognized as contributing risk factors to the dietary plan.^26^ On the other hand, Mansouri et al. showed that high socioeconomic status can lead to CC in some areas of Tehran, because of consuming unhealthy diets, such as fast foods and high‐fat diets, and lower physical mobility.^18^

In this study, the prevalence of some noninfectious risk factors including diet and lifestyle affecting the disease was determined in all provinces of Iran. The results showed that the highest incidence of CC was in Yazd and Guilan provinces. Several studies in the United States and Europe have recognized environmental and behavioral factors associated with CC.^27^, ^28^, ^29^

A systematic analysis conducted by the World Cancer Research Fund International (2020) revealed that factors including obesity, alcoholic beverages, low physical activity, and adopting western eating habits contribute to the development of CC. While eating fiber such as fruits and vegetables, dairy products, and physical activity can reduce the illness.^30^ Other reports have also suggested that lifestyle and a healthy diet (such as fruits, vegetables, whole grains, legumes, seafood, and dairy products) can prevent CC.^29^, ^30^, ^31^

The OLS analysis of the research data introduced tobacco and cigarette smoking as the main risk factors that were significantly associated with CC incidence (*p* < 0.05). However, there was no significant relationship between alcohol consumption, obesity, and fat around the waist and the incidence of CC in Iran. Cigarette smoking is known as the most important factor in lifestyle.^29^, ^32^ Previous studies have confirmed the strong relationship between tobacco and cigarette smoking and cancer. A meta‐analysis showed that the risk of CC in smokers is likely 1.25 times more than in non‐smokers. There was also a significant relationship between CC and smokers above 30 years old.^33^ Furthermore, the risk of development of CC can increase linearly with the intensity and smoking duration.^34^ Another important result of the present study was the existence of a significant relationship between abdominal obesity and the incidence of rectal cancer. This finding has been contradictory in previous studies. A meta‐analysis study.^35^ was conducted in 2007 to investigate the association between obesity and colorectal cancer in observational studies related to epidemiological guidelines. In that study, the information on 70,000 patients with colorectal cancer was examined and identified. It was found that obesity has a direct and independent relationship with this disease. In addition, patients with a BMI of 30 kg/m^2^ have an approximately 20% higher risk of developing colorectal cancer than those with a normal BMI (less than 25). Moreover, for every 2 cm increase in waist circumference (as a measure of abdominal/central obesity), the risk of this cancer increases by 4%. However, in our study, general obesity was not significantly associated with rectal cancer. Another systematic review and meta‐analysis study^36^ was conducted in 2017 to evaluate abdominal obesity (regarding waist‐to‐hip ratio) and colorectal cancer risk. In this study, 19 cohort studies and a total of 12,837 patients with colorectal cancer were studied. The results having been analyzed showed that abdominal obesity was significantly associated with colorectal cancer, and with each of the colon and rectum cancers separately. The finding is consistent with the results of our study. Confirming the findings of our study, Seo et al. showed that obesity increases the risk of colorectal cancer in men and women.^37^ Also, the results of another study showed that the implementation of smoking cessation programs in the EMRO countries is necessary because there is a positive relationship between smoking and CRC.^38^ It seems that the implementation of the screening program for obese people as well as people with tobacco use in Iran can detect the disease in the early stages and increase the survival of these patients.

Another study^39^ conducted in China in 2013 on 134,255 patients with colorectal cancer and its relationship with their body weight and body fat distribution showed that there is no significant relationship between abdominal obesity and general rectal cancer. Although this study found a significant relationship between abdominal obesity and colon cancer in men, its findings do not conform to those of our study. Due to the sparseness of the information, we preferred to use total data and we could not analyze data in the age and gender sub‐groups, which is the limitation of the study.

Strength and limitations: the strength of our study, particularly regarding the large sample size obtained from the surveys. A robust sample size enhances the reliability of our findings and allows for a more comprehensive understanding of the dietary and lifestyle factors in the population studied. We also acknowledge that our study has limitations, particularly regarding the lack of detailed sociodemographic variables such as age and gender in our analysis.

## 5 CONCLUSION

In conclusion, the spatial analysis method using geographical weight regression in disease mapping is highly recommended to inform high‐risk areas for colon and rectal cancers. This study showed that CC high‐risk areas are located in central and northern parts of Iran, and the significant risk factors related to CC were found to be tobacco use and cigarette smoking, and for RC was abdominal obesity. These findings are helpful to inform policymakers to plan screening services to reduce CC and RC, especially in high‐risk populations.

## STRENGTH POINTS

As the strength point, we used GIS and GWR models to evaluate colon and rectal cancer risk factors better.

## LIMITATIONS OF THE STUDY

We used the information for 2016 Steps that was on the vizit website (https://vizit.report/en/index.html) and the information for 2019 was not available due to non‐publication.

## AUTHOR CONTRIBUTIONS

**Zahra Montaseri:** Conceptualization; Writing—original draft. **Hossein Kargar:** Data curation; Writing—original draft; Writing—review and editing. **Mehdi Sharafi:** Conceptualization; Methodology; Software; Supervision; Writing—original draft. **Sima Afrashteh:** Software; Writing—original draft; Validation.

## CONFLICT OF INTEREST STATEMENT

The authors declare no conflicts of interest.

## ETHICS STATEMENT

In this study, ecological data has been used. This study was approved by the Ethics Committee of Fasa University of Medical Sciences (IR. FUMS. REC.1400.054)

## TRANSPARENCY STATEMENT

The lead author Mehdi Sharafi affirms that this manuscript is an honest, accurate, and transparent account of the study being reported; that no important aspects of the study have been omitted; and that any discrepancies from the study as planned (and, if relevant, registered) have been explained.

## Data Availability

The data that support the findings of this study are openly available in STEPS at https://vizit.tums.ac.ir/panel/steps-2020/en/main.html#/forestLocation. Data are available at: https://vizit.report/en/index.html. “All authors have read and approved the final version of the manuscript. The corresponding author had full access to all of the data in this study and takes complete responsibility for the integrity of the data and the accuracy of the data analysis.”
